# The other side of opioid receptor signaling: regulation by protein-protein interaction

**DOI:** 10.1186/2193-1801-4-S1-L21

**Published:** 2015-06-12

**Authors:** Zafiroula Georgoussi

**Affiliations:** Institute of Biosciences and Applications, Laboratory of Cellular Signaling and Molecular Pharmacology, National Centre for Scientific Research “Demokritos”, Greece

**Keywords:** Opioid receptors, RGS proteins, STAT5

Opioid receptors (OR) are widely known as mediators of the analgesic effects of opioids and also contribute to the development of tolerance and dependence. Moreover, opioids are implicated in cell proliferation and survival. How opioids modulate these downstream signaling pathways is a research receiving a lot of attention. Our group aims to define the signaling pathways through which opioid receptors participate in these physiological processes. Emphasis is given to unconventional interacting partners of the μ and δ-opioid receptors such as the Regulators of G protein signalling (RGS) proteins and STAT5B (Georgoussi et al. 2012). Evidence will be presented with which RGS proteins opioid receptors interact, how RGS members confer selectivity to receptors to choose a specific subset of G proteins, how activation of opioid receptors result in recruitment of RGS proteins to the plasma membrane and exert a differential modulatory effect in ERK1,2 phosphorylation, agonist-driven adenylyl cyclase inhibition and internalization of the opioid receptors (Papakonstantinou et al. 2015). Moreover evidence will be presented on how STAT5B associates with the δ-opioid receptor and forms selective pairs with selective Gα, Gβγ subunits and RGS proteins, and how activation of the δ-opioid receptor with selective agonists promotes a multi-component signaling complex involving the STAT5B transcription factor and other signaling intermediates to mediate neuronal survival and neurite outgrowth (Georganta et al.2013). Understanding the mechanism that control OR signaling is important to address problems related to phenomena such as pain perception, tolerance and dependence that occur upon chronic opiate administration and define whether disruption of such interactions may contribute to the development of novel therapeutic strategies.

